# The top 100 Twitter influencers in cardiology

**DOI:** 10.3934/publichealth.2021058

**Published:** 2021-10-26

**Authors:** Onoriode Kesiena, Henry K Onyeaka, Setri Fugar, Alexis K Okoh, Annabelle Santos Volgman

**Affiliations:** 1 Department of Internal Medicine, Piedmont Athens Regional Medical Center, Athens, USA; 2 Department of Psychiatry, Massachusetts General Hospital/Mclean, Boston, USA; 3 Division of Cardiology, Department of Internal Medicine, Rush University Medical Center, Chicago, USA; 4 Division of Cardiology, Cardiovascular Research Unit, RWJ Barnabas Health, NBIMC, Newark, New Jersey, USA

**Keywords:** Twitter, cardiology, influence, *h-index*

## Abstract

**Importance:**

Twitter represents a growing aspect of the social media experience and is a widely used tool for public education in the 21^st^ century. In the last few years, there has been concern about the dissemination of false health information on social media. It is therefore important that we assess the influencers of this health information in the field of cardiology.

**Objective:**

We sought to identify the top 100 Twitter influencers within cardiology, characterize them, and examine the relationship between their social media activity and academic influence.

**Design:**

Twitter topic scores for the topic search “cardiology” were queried on May 01, 2020 using the Right Relevance application programming interface (API). Based on their scores, the top 100 influencers were identified. Among the cardiologists, their academic h-indices were acquired from Scopus and these scores were compared to the Twitter topic scores.

**Result:**

We found out that 88/100 (88%) of the top 100 social media influencers on Twitter were cardiologists. Of these, 63/88 (72%) were males and they practiced mostly in the United States with 50/87 (57%) practicing primarily in an academic hospital. There was a moderately positive correlation between the *h-index* and the Twitter topic score, *r* = +0.32 (*p*-value 0.002).

**Conclusion:**

Our study highlights that the top ranked cardiology social media influencers on Twitter are board-certified male cardiologists practicing in academic settings in the US. The most influential on Twitter have a moderate influence in academia. Further research should evaluate the relationship between other academic indices and social media influence.

## 1. Introduction

Social media platforms such as Twitter using posts called tweets have altered the way people disseminate information [1]. Sharing of information is made possible by the interactions a tweet generates among users. As compared to a regular user, tweets from a small proportion of users called influencers tend to generate the most interactions. These influencers typically have a large online following and may or may not be experts in the issue of discourse [2],[3]. With the help of these influencers, health information may be communicated to a large audience promptly in situations where it is necessary to do so.

Studies have reported health-related benefits from the use of social media platforms such as Twitter as well as a concern. It has been reported as an excellent place to discover current topics of discourse about vaccines and also to promote vaccination [4]. By using semantic analysis to identify influencers on Twitter, vaccine-hesitant communities can be identified and targeted for inventions. Perhaps as a platform for information dissemination about health, interactions on Twitter can positively influence users by improving their health-seeking behaviors. They can then become aware of the right source of information and seek the right remedy for their health conditions [5],[6].

However, sometimes, it is unclear which individuals are influencing these interactions. Given the potential that exists for the dissemination of inaccurate health information [7], there is a need to have experts at the forefront of information dissemination on this platform. Cardiovascular health is an area in which interactions that can lead to a positive health-seeking behavior is needed. This need is made obvious by the growing burden of cardiovascular diseases despite the traditional efforts from various stakeholders [8]. As experts, cardiologists can increase awareness, build partnerships and act as advocates of cardiovascular health in their roles as Twitter influencers [9],[10].

Traditionally cardiologists are considered experts by their years of experience and their research output. This research output can be measured by different matrixes, one of which is the *h-index* [Hirsch index—productivity in terms of number of publications and impact (number of citations) of the publication] [11]. One would expect that the most influential cardiologists on Twitter also have the highest research output, but this may not be the case. It will also be interesting to see if the most influential Twitter users in the field of cardiology experts are indeed, in this case physicians. The goal of this study therefore, is to assess the top influencers in the field of cardiology who are actively influencing information dissemination on Twitter and to assess if there is any correlation between the Twitter influence and academic influence of the practicing cardiologists.

## 2. Methods

On May 01, 2020, similar to the method used in other studies [12],[13], the Right Relevance Application Programming Interface (API) (www.rightrelevance.com, San Francisco, CA, United States) was queried using the search word “cardiology”. The API generated a Twitter topic score for “cardiology”. This score is a measure of how much interactions from other users an influencer earns from a tweet about a topic in the field of cardiology. Subsequently, a rank list of the top 100 cardiology Twitter influencers with their Twitter handles, Twitter names, Twitter profiles, and the number of followers was generated. We excluded handles belonging to organizations as the study's focus was on individual users. Individuals were characterized by sex, duration in years post fellowship training, occupation, area/field of focus for those who were cardiologist physicians, practice setting (academic hospital practice, academic & private hospital practice, non-academic hospital practice, private hospital practice, and both hospital practice & entrepreneurship), and location. These characteristics were identified on their Twitter profiles and web sources such as Doximity (San Francisco, CA, United States), LinkedIn (Sunnyvale, CA, United States), ResearchGate (Berlin, Germany), and practice and institutional websites. The *h-index* scores of the top cardiologist influencers were obtained using Scopus (Reed Elsevier, London, United Kingdom) on May 07, 2020, and added to the database to represent their academic influence. The median *h-index* of the influencers that were cardiologists was calculated and a Pearson correlation was performed between the *h-indices* of the cardiologists and their Twitter topic score to evaluate the relationship. Statistics and graphical representation were performed in Microsoft Excel (Seattle, WA, United States).

## 3. Results

**Figure 1.**
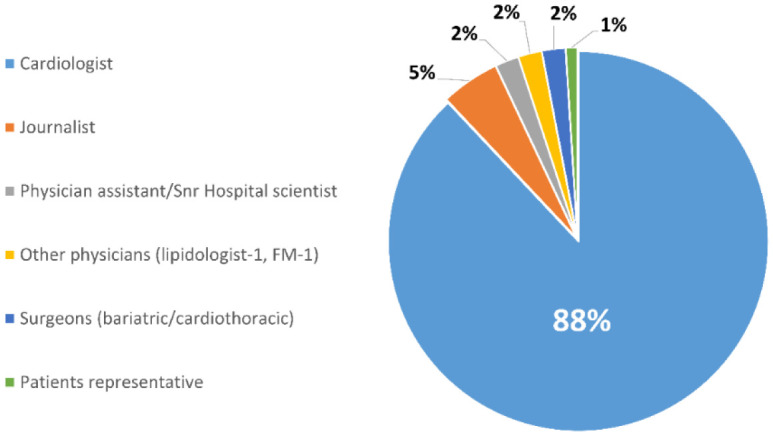
Percent distribution of the top influencers in the field of cardiology.

The top 100 most influential individuals in cardiology on Twitter were evaluated (Table 1). Males made up 70 (70%) of the influencers while 30 (30%) were females. Eighty-eight (88%) of the top influencers were cardiologists; 5 (5%) were journalists; 2 (2%) were surgeons (bariatric and cardiothoracic surgeons); 2 (2%) were other physicians (Family medicine physician and a Lipidologist); 2 (2%) consisted of a physician assistant and a senior hospital scientist, and 1 (1%) was a representative for cardiology patients (Figure 1). Eighty-eight (88%) of influencers worked in the United States and 12 (12%) worked outside the United States. In the US, the most common locations in which they worked include Massachusetts 12/88 (13%) and California 11 (13%). Outside the United States, the most common locations included the United Kingdom 4/12 (33%) and Canada 3/12 (25%) (Table 2).

**Table 1.** API generated ranking of the top 100 influential individuals in cardiology on Twitter.

Rank	Twitter handle	Twitter name	Post-fellowship duration (years)	Occupation
1.	cmichaelgibson	Michael C. Gibson	27	Interventional cardiologist
2.	erictopol	Eric Topol	35	Cardiologist-scientist
3.	drpascalmeier	Pascal Meier	20	General cardiologist
4.	drmarthagulati	Martha Gulati	19	Preventive cardiologist
5.	drjohnm	John Mandrola	25	General cardiologist
6.	heartotxheartmd	John P Erwin III	22	General cardiologist
7.	heartbobh	Robert Harrington	27	Interventional cardiologist
8.	drsethdb	Seth Bilazarian	27	Interventional cardiologist
9.	hmkyale	Harlan Krumholz	28	General cardiologist
10.	drsheilasahni	Sheila Sahni	3	Interventional cardiologist
11.	cardiobrief	Larry Husten	N/A	Medical journalist
12.	dlbhattmd	Deepak L. Bhatt	20	Interventional cardiologist
13.	gina_lundberg	Gina Lundberg	26	Preventive cardiologist
14.	mwaltonshirley	Melissa Walton-Shirley	29	General cardiologist
15.	erinmichos	Erin D. Michos	13	Preventive cardiologist
16.	ajaykirtane	Ajay Kirtane	14	Interventional cardiologist
17.	shelleywood2	Shelley Wood	N/A	Medical journalist
18.	greggwstone	Gregg W. Stone	31	Interventional cardiologist
19.	rwyeh	Robert W. Yeh	10	General cardiologist
20.	svraomd	Sunil V. Rao	16	Interventional cardiologist
21.	drtoniyasingh	Toniya Singh	17	General cardiologist
22.	docsavagetju	Michael Savage	35	Interventional cardiologist
23.	drlaxmimehta	Laxmi Mehta	14	Preventive cardiologist
24.	keaglemd	Kim Eagle	34	General cardiologist
25.	minnowwalsh	Minnow Walsh	21	Cardiologist non-invasive imaging
26.	drkevincampbell	Kevin Campbell	17	Cardiologist-electrophysiology
27.	heartdocsharon	Sharon Mulvagh	31	Cardiologist non-invasive imaging
28.	drroxmehran	Roxana Mehran	25	Interventional cardiologist
29.	nmhheartdoc	Clyde Yancy	31	General cardiologist
30.	willsuh76	William Suh	10	Interventional cardiologist
31.	drjmieres	Jennifer Mieres	28	Cardiologist non-invasive imaging
32.	chadialraies	Chadi Alraies	4	Interventional cardiologist
33.	samrrazamd	Sam Raza	2	Cardiologist non-invasive imaging
34.	venkmurthy	Venk Murthy	8	Cardiologist non-invasive imaging
35.	arh_cardio	Andrew R. Houghton	14	Cardiologist non-invasive imaging
36.	sharonnehayes	Sharonne Hayes	30	Preventive cardiologist
37.	pamelasdouglas	Pamela S Douglas	36	Cardiologist non-invasive imaging
38.	cpcannon	Christopher Cannon	20	General cardiologist
39.	drlindamd	Linda Girgis	N/A	Family medicine physician
40.	ejsmd	Edward J Schloss	23	Cardiologist-electrophysiology
41.	fischman_david	David L. Fischman	29	Interventional cardiologist
42.	ankurkalramd	Ankur Kalra	3	Interventional cardiologist
43.	doctorwes	Westby Fisher	22	Cardiologist-electrophysiology
44.	califf001	Robert M Califf	38	General cardiologist
45.	vietheartpa	Viet Le	16	Cardiology-physician assistant
46.	tctmd_yael	Yael L. Maxwell	N/A	Medical journalist
47.	drdave01	David E. Albert	39	Cardiologist-entrepreneur
48.	pooh_velagapudi	Poonam Velagapudi	2	Interventional cardiologist
49.	anastasiasmihai	Anastasia S Mihailidou	N/A	Senior hospital scientist
50.	cpgale3	Chris P Gale	6	General cardiologist
51.	majazayeri	Ali Jazayeri	0	Cardiology fellow
52.	nihdirector	Francis S. Collins	36	General cardiologist-scientist
53.	sethjbaummd	Seth J. Baum	30	Interventional cardiologist
54.	drraviele	Raviele Antonio	46	Cardiologist-electrophysiology
55.	leftbundle	Mintu Turakhia	12	Cardiologist-electrophysiology
56.	lipiddoc	James Underberg	N/A	Lipidologist
57.	richardbogle	Richard Bogle	13	Interventional cardiologist
58.	michaeltctmd	Michael O'Riordan	N/A	Medical journalist
59.	jgrapsa	Julia Grapsa	7	Cardiologist non-invasive imaging
60.	ethanjweiss	Ethan Weiss	17	Preventive cardiologist
61.	neilflochmd	Neil Floch	N/A	Bariatric surgery
62.	davidmaymd	David May	32	Interventional cardiologist
63.	herbaronowmd	Herb Aronow	17	Interventional cardiologist
64.	drryanpdaly	Ryan P. Daly	10	Cardiologist non-invasive imaging
65.	skathire	Sek Kathiresan	12	Preventive cardiologist/entrepreneur
66.	cardiacconsult	Jordan Safirstein	12	Interventional cardiologist
67.	pnatarajanmd	Pradeep Natarajan	5	Preventive cardiologist
68.	debbemccall	Debbe McCall	N/A	Patient research/representative
69.	davidlbrownmd	Clinically Conservative Cardiologist	27	Interventional cardiologist
70.	jjheart_doc	James Januzzi	20	Cardiologist non-invasive imaging
71.	onco_cardiology	Juan Lopez-Mattei	7	Cardio-oncologist
72.	drjohndaymd	John Day	20	Cardiologist-electrophysiology
73.	aalahmadmd	Amin Al-Ahmad	17	Cardiologist-electrophysiology
74.	toddneale	Todd Neale	N/A	Medical journalist
75.	josejgdnews	Jose Juan Gomez	25	Cardiologist non-invasive imaging
76.	jonhsumd	Jonathan Hsu	7	Cardiologist-electrophysiology
77.	mkittlesonmd	Michelle Kittleson	15	Heart transplant cardiologist
78.	lisarosenbaum17	Lisa Rosenbaum	8	Interventional cardiologist
79.	toaster_pastry	Wayne Whitwam	14	Cardiologist-electrophysiology
80.	avolgman	Annabelle Volgman	30	Cardiologist-electrophysiology
81.	rblument1	Roger Blumenthal	28	Preventive cardiologist
82.	achoiheart	Andrew D. Choi	10	Cardiologist non-invasive imaging
83.	mgkatz036	Michael Katz	5	Cardiologist-electrophysiology
84.	prashsanders	Prashanthan Sanders	17	Cardiologist-electrophysiology
85.	bcostellomd	Briana Costello	0	Interventional cardiologist
86.	popmajeffrey	Jeffrey Popma	30	Interventional cardiologist
87.	adribaran	Adrian Baranchuk	23	Cardiologist-electrophysiology
88.	sandylewis	Sandra Lewis	37	General cardiologist
89.	yadersandoval	Yader Sandoval	3	Interventional cardiologist
90.	drquinncapers4	Quinn Capers	21	Interventional cardiologist
91.	dramirkaki	Amir Kaki	11	Interventional cardiologist
92.	jamesbeckerman	James Beckerman	14	Genera cardiologist
93.	eirangorodeski	Eiran Gorodeski	11	General cardiologist
94.	docstrom	Jordan Strom	3	Cardiologist non-invasive imaging
95.	dbelardomd	Danielle Belardo	0	Cardiology Fellow
96.	sergiopinski	Sergio Pinski	27	Cardiologist-electrophysiology
97.	arieblitzmd	Arie Blitz	N/A	Cardiothoracic surgeon
98.	ash71us	Ashish Aneja	8	General cardiologist
99.	tjaredbunch	Thomas Jared Bunch	12	Cardiologist-electrophysiology
100.	rfredberg	Rita Redberg	32	General cardiologist

**Table 2.** Practice location of the top 100 most influential individuals.

United States	Percentage	International	Percentage
Massachusetts	13.64%	United Kingdom	33.33%
California	12.50%	Canada	25.00%
Texas	7.95%	Switzerland	8.33%
New York	7.95%	South wales	8.33%
Ohio	6.82%	Italy	8.33%
New Jersey	4.55%	Spain	8.33%
Connecticut	3.41%	Australia	8.33%
Baltimore	3.41%		
North Carolina	3.41%		
Pennsylvania	3.41%		
Michigan	3.41%		
Illinois	3.41%		
Utah	3.41%		
Kentucky	2.27%		
Missouri	2.27%		
Indiana	2.27%		
Minnesota	2.27%		
Kansas	2.27%		
Florida	2.27%		
Oregon	2.27%		
Arizona	1.14%		
Georgia	1.14%		
Nebraska	1.14%		
Rhode Island	1.14%		
Washington	1.14%		
Wisconsin	1.14%		

**Figure 2.**
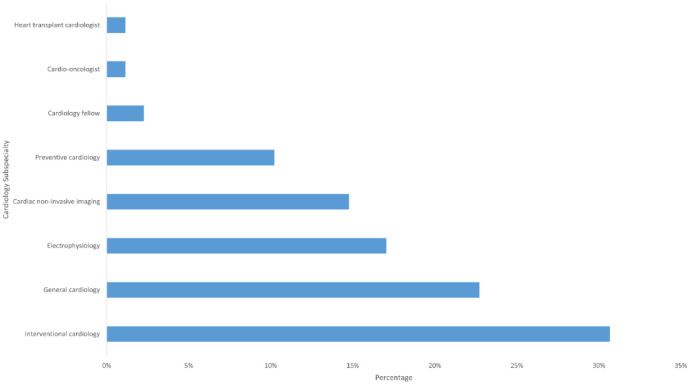
Distribution of cardiologist by specialty.

Approximately 63/88 (72%) of the top influencers that were cardiologists were males and 25/88 (28%) were females. Of the 88 cardiologists, 87 were actively practicing. Of the practicing cardiologists, about 50/87 (57%) of them worked primarily in an academic hospital setting, 33/87 (38%) in non-academic hospitals, 2/87 (2%) in both academic & private facilities, 1/87 (1%) in private hospitals alone, and 1/87 (1%) worked both in a non-academic hospital and as an entrepreneur. As shown in Figure 2, Twenty-seven (31%) of cardiologist influencers were focused in interventional cardiology, 20/88 (23%) in general cardiology, 15/88 (17%) in electrophysiology, 13/88 (15%) in cardiac non-invasive imaging and 9/88 (10%) in preventive cardiology.

The median and mean *h-index* of the top influencers who were cardiologists was 22 (interquartile range = 32.5) and 41.84 ± 9.89 (mean ± 95% CI) respectively. There was a moderately positive correlation between their Twitter topic score and *h-index*, *r* = +0.32 (*p*-value 0.002).

## 4. Discussion

The study aimed to assess the top individuals driving the discussions in cardiology on Twitter and to analyze if they were as influential in academia as they were on Twitter. We found out that the top 100 Twitter influencers were male cardiologists in the United States with 30% women, they work in academic hospitals and interventional cardiologists represent the largest proportion of cardiologists among the influencers. In addition, there was a moderately positive correlation between their academic and Twitter influence.

Most of the top 100 cardiology Twitter influencers were US cardiologists. This made up about 85% of the total population studied. They also practice mostly in academic institutions. These individuals are currently influencing the engagements in the field of cardiology on Twitter, and it is consistent with findings from other studies. These other studies evaluated the top influencers in other medical fields on Twitter and found them to be experts in these fields [12],[13]. This is important given that people are more likely to engage a post on Twitter when experts lead the discussion [14]. However, this may not be enough to prevent the dissemination of false information which leads to public mistrust [14],[15], as among the top 100, 12% were non-cardiologists and may be considered as non-experts.

We also found out that among the influencers that were cardiologists, 2 out of 3 were males. This mostly can be attributed to the small percentage of women who are currently cardiologists [16]. A recent study reported that despite the high percentage of female internal medicine residents, only about 13% of cardiologists are women [17]. Although it is not surprising that females are a minority, with 1 in 3 cardiology influencers being females, it however shows a larger representation of female cardiologists on Twitter which doubles the current trend in the US. There also seems to be a flattening of the hierarchy with a mix of early career (e.g., Briana Costello, Sam Raza), mid-career (e.g., William Suh, Andrew R. Houghton) and advanced stage career (e.g., Michael Gibson, Martha Gulati) professionals being among the top influencers. A positive finding given the criticism the historical hierarchy in medicine has received in recent years [18].

With regards to their location, only a few cardiologists outside the US were part of the top 100 cardiology influencers. This may be attributed to reports of anti-social media policies in some European countries [19] and the resultant low adoption rates of social media platforms [20],[21]. This may account for why fewer cardiologists outside the US are currently in the top 100 influencers on Twitter. Nevertheless, findings of the massive use of Twitter during European conferences to share impressions have been reported [22],[23]. In addition, Twitter has been reported as a source of data in the research of noncommunicable diseases in European studies [24]. These reports are inconsistent with the reported anti-social media policies outside the US and there may be other reasons behind these findings.

In addition, we found out that most of the top cardiologist influencers practice in academic hospitals. Studies have shown a high research output from cardiologists who practice in this setting as compared to those who practice in non-academic settings [25]. This is due to the heavy emphasis on research in academic hospitals as compared to non-academic hospitals. These cardiologists have also been found to be more likely to tweet about conferences, research activities, and meetings they attend [26] as compared to those in non-academic settings.

With regards to the overall academic influence, the median *h-index* of the top cardiologist influencers (median *h-index*, 22) found in our study was higher than that of the orthopedic (median *h-index*, 7) and plastic surgeons (median *h-index*, 5) in studies done in 2018 and 2019 respectively [12],[13]. In a comparison of the median *h-index* and their Twitter influence, there was a moderately positive correlation between the two. The moderate positive relationship implies that not only are these top influential cardiologists more active in research as compared to other specialties, they are also almost as influential on Twitter as they are in academia. The most active influential cardiologists may be tweeting more about breakthroughs in cardiovascular research [27]. This is relevant as social media has become a tool to reach millions of people and gather data, and as such, physicians need to be conversant and active in its use. Twitter is a tool to promote and direct attention to specific research topics [28] and was found to be an effective way to increase citations of a publication, influencing the *h-index* of an author [29].

This study has a few strengths. First, the large sample size of this study allowed for adequate characterization of the influencers. Second, we used the Right Relevance API which has successfully been used to mine data from Twitter for other studies. Third, the academic influence was computed using the *h-index*, a scoring system that shows a high correlation with other variants [30]. Despite the strengths of this study, it has some limitations worthy of note. First, the data was made of incomplete Twitter profiles that had to be completed using sources such as Doximity and LinkedIn. Second, a different API using another algorithm may generate a data set entirely different from this data set. Third, there are other social media platforms apart from Twitter where other cardiologists may be more active such as Facebook and Instagram. Lastly, the h-index pays attention only to h-core papers, ignores most papers with a low citation frequency, and lacks sensitivity to highly cited papers.

In conclusion, our study showed that when examining the influential voices in cardiology on Twitter, there is a broad range of sub-specialties represented, with interventional cardiologists being the most prominent. There was a geographical diversity as well as a flattening of the hierarch, with a mix of early career (e.g., Briana Costello, Sam Raza), mid-career (e.g., William Suh, Andrew R. Houghton) and advanced stage career (e.g., Michael Gibson, Martha Gulati) professionals. Thirty percent were women, which more than doubles the number of women estimated to be practicing cardiovascular medicine. This reflects the challenges that remains in closing the gender gap between men and women as influencers in cardiovascular medicine. These influencers were as influential in the academia as they are on Twitter. Future studies should exam the contents of the posts made by these influencers and also consider other indexes of academic influence like g-index, AR-index, p-index, and integrated impact indicator or academic trace as they relate to social media influence.
